# Influence of Anti-Coccidial Compounds and Phytogenic Saponin Extracts on In Vitro and In Vivo Ruminal Fermentation and Methane Production of Cattle

**DOI:** 10.3390/ani13142308

**Published:** 2023-07-14

**Authors:** Ronald J. Trotta, Kelly K. Kreikemeier, Scott Foote, Kyle R. McLeod, David L. Harmon

**Affiliations:** 1 Department of Animal and Food Sciences, University of Kentucky, Lexington, KY 40546, USAkyle.mcleod@uky.edu (K.R.M.); 2 Hoxie Feedyard, Foote Cattle Co., Hoxie, KS 67740, USA

**Keywords:** amprolium, decoquinate, feed additive, feedlot nutrition, monensin, ruminal digestion, terpenes, *Quillaja saponaria* extract, *Yucca schidigera* extract

## Abstract

**Simple Summary:**

There is rising interest globally in reducing the use of antibiotics in livestock feeding regimens. Phytogenic feed additives have been evaluated for their potential use to replace compounds such as ionophores in finishing cattle diets. The effects of saponin-containing extracts from *Yucca schidigera* (YSE) on ruminal fermentation, average daily gain, and feed efficiency appear to be similar to monensin; however, YSE is not approved for the control or prevention of coccidiosis in cattle. Therefore, the objectives of this study were to evaluate sources and levels of anti-coccidial compounds and saponins, individually or in combination, on in vitro and in vivo ruminal fermentation and CH_4_ production. In summary, the combination of decoquinate with YSE did not influence in vivo CH_4_ production but tended to increase ruminal propionate proportion. Monensin inclusion decreased in vitro CH_4_ production and the acetate:propionate. Increasing saponin inclusion increased the propionate proportion in vitro but was not accompanied by a reduction in CH_4_ production.

**Abstract:**

Four experiments were conducted to evaluate sources of anti-coccidial compounds and phytogenic saponin extracts on in vitro and in vivo ruminal fermentation and CH_4_ production at multiple inclusion levels. In experiment 1, eight steers were fed either a finishing diet or a finishing diet supplemented with 0.5 mg/kg BW decoquinate (DCQ) and 3.33 mg/kg BW *Yucca schidigera* extract (YSE), and respiratory gas exchange was measured. In experiment 2, four ruminally-cannulated steers were fed the same treatments as experiment 1, and ruminal fermentation was evaluated. Anti-coccidial sources (experiment 3; monensin, DCQ, amprolium) and saponin sources (experiment 4; YSE, *Quillaja saponaria* extract) and levels were evaluated for effects on in vitro ruminal fermentation and CH_4_ production. DCQ + YSE supplementation did not influence (*p* ≥ 0.24) in vivo respiratory gas consumption/production, in situ DM degradation, or liquid passage kinetics. Ruminal propionate proportion tended to increase (*p* = 0.09) with DCQ + YSE. Monensin decreased (*p* ≤ 0.04) in vitro acetate:propionate and CH_4_ production; saponin supplementation linearly increased (*p* < 0.01) propionate proportion but did not influence (*p* ≥ 0.38) in vitro CH_4_ production. Saponins and non-antibiotic anti-coccidials did not influence in vitro or in vivo CH_4_ production with finishing diets.

## 1. Introduction

Ruminal methanogenesis is a potential pathway for the elimination of H_2_ and the regeneration of NAD^+^ for microbial glycolysis [1]. Methane mitigation strategies can largely be classified into three main groups: animal and feed management, diet formulation, and rumen manipulation [2]. High-concentrate diets containing moderate to large proportions of starch are fed to increase the energy density of the diet to allow for more efficient growth and improved product quality. When high-concentrate diets are fed to beef cattle, the metabolizable energy to digestible energy ratio increases compared to feeding high-roughage diets [3]. The net result is proportionally lower CH_4_ energy losses when feeding high-concentrate diets [3]. Although feeding high-concentrate diets results in less CH_4_ production per unit of fermentable organic matter compared with high-roughage diets, the challenge remains to develop strategies to further decrease enteric CH_4_ emissions from feedlot cattle. Thus, combining enteric CH_4_ mitigation strategies that target animal and feed management, diet formulation, and rumen manipulation simultaneously could potentially be effective for achieving additive reductions in enteric CH_4_ emissions [2,4].

Ionophores, such as monensin, have historically been supplemented in finishing cattle diets to improve average daily gain, feed efficiency, and reduce CH_4_ emissions [5]. Dietary monensin inclusion leads to energetic advantages by modifying ruminal microbial populations, which results in increased propionate proportion and decreased CH_4_ production [6]. However, there is rising global interest in removing antibiotics, such as ionophores, from livestock diets, and consequently, phytogenic feed additives have been explored for their potential use in ruminant livestock diets [7,8,9,10]. Recent studies have demonstrated that *Yucca schidigera* extract (YSE) supplementation increased the average daily gain and feed efficiency of finishing cattle when included in the diet at up to 4 g/d [11,12]. In ruminants, isolated saponins or saponins in phytogenic extracts, such as YSE, have been shown to decrease CH_4_ production both in vitro [13,14] and in vivo [15,16]. Potential mechanisms by which YSE decreases CH_4_ production include altering ruminal microbial populations, inhibiting ruminal H_2_ production, or decreasing feed intake or ruminal digestibility [14]. Lila, et al. [15] found that feeding sarsaponin to steers at 0.5% or 1% of the diet (11.2 or 22.4 g/d) on a dry matter (DM) basis decreased in vivo CH_4_ production by up to 12.7% and was associated with increased ruminal propionate proportion and decreased ruminal NH_3_ and protozoa concentrations. Those authors also found that dietary sarsaponin inclusion decreased total-tract DM and neutral detergent fiber digestibility [15]. In general, the effects of YSE on ruminal fermentation, growth performance, and feed efficiency appear to be similar to the effects of ionophores in cattle diets [17].

Monensin functions as an anti-coccidial compound [18,19], and although YSE has some anti-coccidial activity [17], its potential application in the prevention or control of coccidiosis in finishing cattle diets has not yet been determined [20]. Decoquinate (DCQ) is a non-antibiotic feed additive approved for use in the control of coccidiosis in cattle in multiple countries worldwide [21]. Increasing DCQ inclusion in high-forage and high-concentrate diets did not influence diet digestibility or ruminal fermentation characteristics [22], but it is thought that increased average daily gain and feed efficiency of beef cattle supplemented with DCQ is because of its function as a cocciodiostat [21,22,23,24]. The objectives of the current study were to evaluate the combination of YSE with DCQ on CH_4_ production of steers using indirect calorimetry and ruminal fermentation characteristics. We hypothesized that feeding YSE in combination with DCQ would decrease in vivo CH_4_ production, and increase ruminal propionate proportion, without influencing the rate or extent of in situ ruminal DM degradability. We further evaluated sources of anti-coccidial compounds (monensin vs. non-antibiotic) and phytogenic saponin extracts (steroidal vs. triterpenoidal) individually for their effects on in vitro ruminal fermentation and CH_4_ production at inclusion levels resembling practical feeding conditions or supranutritional levels.

## 2. Materials and Methods

All animal procedures were approved by the University of Kentucky Animal Care and Use Committee (Protocol 2020-3546).

### 2.1. Experiment 1

Eight Holstein steers (initial body weight (BW) = 309 ± 28.0 kg) were used to determine the effects of DCQ + YSE supplementation on in vivo CH_4_ production. Steers were housed in individual pens (3 m × 3 m) in the Intensive Research Building of the University of Kentucky C. Oran Little Research Center in Versailles, KY, USA. The experimental design was a randomized complete block crossover design with two periods. Steers were fed either the basal diet (control) or the basal diet supplemented with 0.5 mg of DCQ per kg of BW (Deccox; Zoetis, Parsippany, NJ, USA) + 3.33 mg of YSE per kg of BW (30% solids; Micro-Aid Feed Grade Concentrate; DPI Global, Porterville, CA, USA) (DCQ + YSE). The amount of DCQ fed was for the prevention of coccidiosis in ruminating and non-ruminating calves (including veal calves) and cattle caused by *Eimeria bovis* and *E. zuernii*, according to the manufacturer (Zoetis, Parsippany, NJ, USA). The amount of YSE fed was based upon the manufacturer’s recommendation for beef cattle to supply 1 g/d YSE (DPI Global, Porterville, CA, USA).

The basal high-concentrate diet (Table 1) was formulated to supply two times the net energy required for maintenance (NE_m_) and to exceed requirements for ruminally degradable protein, metabolizable protein, vitamins, and minerals [25]. The basal diet was mixed in one batch, vacuum-sealed in Cryovac barrier bags (0.559 m × 0.914 m; Sealed Air Corporation, Charlotte, NC, USA), and frozen at −4 °C until use. Rations were fed (7.09 ± 0.492 kg DM) once daily at 0800. Fine-ground corn (454 g) was mixed with treatments and fed 15 min before the morning feeding each day to ensure complete consumption of the offered dose of DCQ + YSE. Steers had ad libitum access to water throughout the experiment. Periods were 10 d in length, including a 7d adaptation to treatments, followed by a 3 d collection period. Because YSE decreased in vitro CH_4_ production after 24 h of fermentation [14], it was assumed that a 7 d adaptation period would be sufficient to observe a response in in vivo CH_4_ production. Previous research has shown the short-term effects of feed additive supplementation on CH_4_ production [26], with decreases in CH_4_ production occurring within 3 to 4 d [27,28]. Respiratory gas exchange was measured over three consecutive 24 h periods from day 8 to day 10. After completion of the collection period, steers were switched to the opposite treatment to begin the next adaptation period.

#### 2.1.1. Feed Analysis

Samples of the basal diet were collected weekly and were analyzed for nutrient composition, including DM, crude protein, neutral and acid detergent fiber, and minerals by the Dairy One Forage Laboratory (Ithaca, NY, USA). Diet samples were partially dried at 60 °C for 4 h in a forced-air oven (NFTA 2.2.1.1.) and then ground to pass a 1 mm screen using a Wiley mill. Dry matter content was determined by oven-drying for 3 h at 105 °C (NFTA 2.1.4). Nitrogen content was analyzed by combustion (AOAC, 2006; method 990.03) using a CN628 Carbon/Nitrogen Determinator (Leco Corporation, St. Joseph, MI, USA). Crude protein was calculated by multiplying the N concentration × 6.25. Acid detergent fiber and neutral detergent fiber concentrations were determined using the filter bag technique (ANKOM Technology Methods 14 and 15, respectively) with an automated fiber analyzer (ANKOM DELTA; ANKOM Technology, Macedon, NY, USA). Samples were digested in 50 mL MARSXPress vessels (CEM Corporation, Matthews, NC, USA) using a MARS 6 Microwave Digestion System (CEM Corporation, Matthews, NC, USA), and mineral concentrations (Ca and P) were determined using inductively coupled plasma-optical emission spectroscopy (iCAP PRO XP ICP-OES; Thermo Fisher Scientific Inc., Beverly, MA, USA).

#### 2.1.2. Respiratory Gas Exchange

Steers were fed treatments for 7 d in individual pens before being transferred to metabolism stalls (1.52 m × 2.13 m) for the measurement of respiratory gas exchange using indirect calorimetry. The design of the head-box-style respiration chambers was previously described by Koontz, et al. [29]. Each respiration chamber was fitted with a waterer, feeder, and air-conditioning unit to maintain consistent temperature (21 °C) and relative humidity (35%). Before use, the zero point of each gas analyzer was calibrated with pure N_2_ gas (American Welding & Gas, Lexington, KY, USA) and the span point of each gas analyzer was calibrated with a custom analytical standard (American Welding & Gas, Lexington, KY, USA; 19.900% O_2_, 0.700% CO_2_, 0.0650% CH_4_). Recovery of O_2_ (105 ± 7.1%) and CO_2_ (102 ± 6.8%) for each respiration chamber was determined by combusting a known amount of propane (119 ± 8.2 g) over a 120 min period.

The flow system and equipment arrangement were similar to those described by Hellwing, et al. [30]. Air flow was maintained at 600 L/min during measures of respiratory gas exchange via a fan motor control (Flow Max XL; Columbus Instruments, Columbus, OH, USA). The airflow from each respiration chamber was measured using mass flow meters (HFM-200; Teledyne Hastings Instruments, Hampton, VA, USA) with laminar flow elements (LS-4F; Teledyne Hastings Instruments, Hampton, VA, USA). Inlet and exhaust airflow from the chambers passed through a 10-channel expansion interface, system sample pump (0.5 L/min), and sample drier before analysis. Sampled air was analyzed for O_2_ concentration by paramagnetic detection (Columbus Instruments, Columbus, OH, USA), and CO_2_ (Columbus Instruments, Columbus, OH, USA) and CH_4_ (VIA-510; Horiba Ltd., Kyoto, Japan) concentrations were measured using infrared gas analyzers. Data from the flow meter and gas analyzers were integrated through a CI-Bus Serial Interface (Columbus Instruments, Columbus, OH, USA), and respiratory gas measurements were recorded in 9 min intervals using Oxymax software (version 4.8.5; Columbus Instruments, Columbus, OH, USA). Oxygen consumption, CO_2_ production, and CH_4_ production were calculated as the total volume of gas consumed/produced after 24 h of measurements. The respiratory quotient was calculated as the liters of CO_2_ produced divided by the liters of O_2_ consumed.

### 2.2. Experiment 2

Four ruminally-cannulated Holstein steers (initial body weight = 469 ± 22.1 kg) were used to determine the effects of DCQ + YSE supplementation on ruminal fermentation, in situ ruminal degradability, and liquid passage rate. The experimental design was a randomized crossover design with two periods. Steers were fed either the basal diet (control) or the basal diet supplemented with 0.5 mg of DCQ per kg of BW + 3.33 mg of YSE per kg of BW (DCQ + YSE). Steers were housed in individual pens (3 m × 3 m), fed the same diet (9.70 ± 0.341 kg DM) once daily, as described in experiment 1, and had ad libitum access to water. Treatments were mixed with finely ground corn, as described in experiment 1. Periods were 14 d in length, including 7 d for adaptation to treatments and 7 d for sample collection. The in situ degradability experiment was conducted from day 8 to day 12, followed by the collection of ruminal fluid on day 14. After completion of the collection period, steers were switched to the opposite treatment to begin the next adaptation period.

#### 2.2.1. In Situ Ruminal Degradability

The in situ ruminal DM degradability of the basal diet was measured using methods previously described [31]. Twenty grams of the basal diet, ground to pass a 2 mm screen, was weighed into nylon bags (10 cm × 20 cm; 50 μm pore size; R1020 Forage Bag; ANKOM Technology, Macedon, NY, USA). The nylon bags were incubated in the rumen for 0, 3, 6, 9, 12, 24, 36, 48, 72, and 96 h. One nylon bag for each incubation timepoint was placed into a zipped wash bag (25 cm × 31 cm; k2107; HomeAide Delicate Wash Bag) that was suspended in the ventral rumen of each steer. Two stainless steel magnets (1.27 cm × 7.62 cm; Silver Star AlniMAX II, Sundown Industries Co., Plainview, NY, USA) were added to each wash bag to ensure immersion in the ventral rumen. Wash bags were attached to a steel chain with a breeching snap clip (2710231; Koch Industries, Inc., Minneapolis, MN, USA). The steel chain was secured to the rumen cannula cap by connecting the steel chain to an inverted U-bolt on the inner portion of the cannula cap (#1 Eazy-out Stopper; Bar Diamond, Inc., Parma, ID, USA) with a breeching snap clip. Wash bags were inserted in reverse order so that all bags were removed from the rumen and rinsed simultaneously. At removal, the wash bags were removed from the steel chain and placed into an ice-water bath to stop fermentation. The nylon bags were removed from the wash bag and rinsed 5 times in a washing machine with 1 min rinse and 2 min spin cycles [32]. The nylon bags were then dried in a 100 °C forced-air oven (1680; Sheldon Manufacturing, Inc., Cornelius, OH, USA) for 48 h to determine in situ DM disappearance.

The potential rate and extent of in situ DM degradation were determined using the first-order asymptotic model [33]:

*y* = *a* + *b* (1 − *e*^−*kd*(*t* − *Lt*)^) (1)

where *y* is the degradation after *t* hours, *a* is the soluble fraction, *b* is the potentially degradable fraction, *k_d_* is the fractional rate of degradation of *b*, *t* is the incubation time (h), and *Lt* is the lag time (h). Dry matter degradability data from times 0, 3, 6, 9, 12, 24, 36, 48, 72, and 96 h were fitted to the above nonlinear model using SAS according to the procedures described by Fadel [34] to generate the parameters described.

The in situ ruminal degradability was determined by using the parameters generated for the rate and extent of degradation, as previously described, and modeled with the rate of passage [35]:

*isRD* = *a* + [(*bk_d_*)/(*k_d_* + *k_p_*)] (2)

where *isRD* is the in situ ruminal degradability, *a* is the soluble fraction, *b* is the potentially degradable fraction, *k_d_* is the fractional rate of degradation of *b*, and *k_p_* is the fractional rate of the liquid passage. The fractional rate of liquid passage measured on day 14 was used for *k_p_*. Degradation coefficients from the generated parameters were converted to percentages by multiplying coefficients by 100.

#### 2.2.2. Ruminal Fermentation and Liquid Passage

On day 14, steers were administered 500 mL of CrEDTA (2.3 g Cr) solution [36] through the rumen cannula 2 h after the morning feeding to evaluate the ruminal liquid passage rate. Approximately 150 mL of ruminal contents were collected from the mid-ventral region of the rumen immediately before administration of the dose (0 h) and at 0.5, 1, 2, 4, 6, 8, 10, 12, 16, 20, and 24 h after dosing. The ruminal contents were squeezed through four layers of cheesecloth to separate the ruminal fluid for analysis of NH_3_ and L(+)-lactate by UV-VIS spectrophotometry and VFA by gas chromatography (Hewlett-Packard 6890 Plus GC; Agilent Technologies Inc., Santa Clara, CA, USA). Squeezed ruminal fluid (5 mL) was combined with 0.5 mL of 500 g/L metaphosphoric acid as a deproteinizing agent [37] and 0.5 mL of 85 mM 2-ethylbutyrate as an internal standard. Acidified samples were frozen at –20 °C to facilitate protein precipitation. Samples were thawed, centrifuged at 20,000× *g* for 15 min at 4 °C, and the supernatant fractions were transferred to autosampler crimp-top vials. Each aqueous sample (0.2 µL) was injected into the inlet with an automatic liquid sampler (7693A; Agilent Technologies Inc., Santa Clara, CA, USA) and vaporization occurred at 260 °C. The sample was carried to the fused silica capillary column (25326; Nukol Capillary GC Column; Supelco Inc., Bellefonte, PA, USA) with He at a 2:1 split ratio. The initial oven temperature was 110 °C for 1 min, ramped to 125 °C at 5 °C per min, ramped to 195 °C at 65 °C per min, and cooled to 110 °C post-detection. Upon column exit, separated SCFA were detected with a flame ionization detector and quantified using electronic integration. Total VFA concentration was considered as the sum of acetate, propionate, isobutyrate, butyrate, isovalerate, and valerate concentration. Molar VFA proportions were calculated as the individual VFA concentration divided by the total VFA concentration and multiplied by 100. Ruminal NH_3_ concentration was analyzed using the glutamate dehydrogenase procedure [38] adapted to a Konelab 20XTi Clinical Analyzer (ThermoFisher Scientific Inc., Beverly, MA, USA). Ruminal L(+)-lactate concentration was measured using the L(+)-lactate dehydrogenase procedure [39,40] adapted to a multi-mode plate reader (BioTek Synergy HTX; Agilent Technologies Inc., Santa Clara, CA, USA).

Chromium concentrations for each sample were determined using atomic absorption spectroscopy (Aanalyst 200; PerkinElmer Inc., Waltham, MA, USA) at a wavelength of 357.87 nm. Baseline concentrations of Cr (0 h) were used to correct the concentrations measured at each time point. The concentration of Cr after dosing and fractional clearance rate of Cr was determined by calculation of the exponential decay rate for Cr using the NLIN procedure of SAS (version 9.4; SAS Institute Inc., Cary, NC, USA) and the following equation:

*Cr_t_* = *Cr*_0_ × *e*^−*kt*^ (3)

where *Cr_t_* represents the Cr concentration at a given time, *Cr*_0_ represents the Cr concentration at time 0-h, *k* represents the fractional rate of Cr clearance which is assumed to be equivalent to the fractional rate of liquid passage (*k_p_*), and *t* represents the time in hours [41,42]. The liquid retention time in the rumen was calculated as the absolute value of 1/*k_p_*. The rumen liquid volume (L) was determined by dividing the amount of Cr dosed by the amount of Cr present at time zero (*C_0_*). The liquid flow rate (L/h) was calculated as *k_p_* × rumen liquid volume.

### 2.3. Experiments 3 and 4

#### 2.3.1. Experiment 3

The objectives of this experiment were to evaluate the effects of antibiotic or non-antibiotic sources of feed additives containing anti-coccidial activity on in vitro CH_4_ production and ruminal fermentation. Additionally, sources of the anti-coccidal compounds were evaluated at multiple inclusion levels that resemble practical feeding conditions or supranutritional inclusion. The experimental design was a randomized complete block design with 7 treatments in a 3 × 2 + 1 factorial arrangement. Three sources of anti-coccidial compounds were tested: monensin (Rumensin 90; Elanco Animal Health Incorporated, Greenfield, IN, USA), DCQ (Deccox; Zoetis Inc., Parsippany, NJ, USA), and amprolium (Corid 9.6% Oral Solution; Huvepharma Inc., Peachtree City, GA, USA). There were two levels for anti-coccidial sources: 1X (based on current feeding recommendations) and 10X (10-fold greater than the 1X dose). The 1X and 10X treatment levels were 30 and 300 mg/kg substrate for monensin, respectively. The 1X and 10X treatment levels were 25 and 250 mg/kg substrate for DCQ, respectively. The 1X and 10X treatment levels were 500 and 5000 mg/kg substrate for amprolium, respectively. Treatment concentrations were corrected for active ingredient percentages and DM content. The basal substrate without any feed additives (0X) was included as a negative control.

Treatments were added to the fermentation vessels according to the proportions described in Table 2. To aid in the accuracy of treatment dispersal, treatment premixes (Rumensin 90 and Deccox) were mixed with the basal substrate. Monensin was prepared as a 10% (*w*/*w*) mixture of Rumensin 90 with the basal substrate. Decoquinate was prepared as 10% and 50% (*w*/*w*) mixtures of Deccox with the basal substrate for the 1X and 10X treatments, respectively. Amprolium was prepared as a 6.4% or 64% (*v*/*v*) solution of Corid 9.6% Oral Solution diluted in water for the 1X and 10X treatments, respectively. All vessels received 500 mg of the basal substrate or basal substrate mixed with treatment so that all flasks contained the same substrate volume. All vessels received 2 mL of water or added treatment diluted in water so that all flasks contained the same liquid volume. There were two replicate fermentation vessels for each treatment, and the experiment was replicated on four separate days.

#### 2.3.2. Experiment 4

The objectives of this experiment were to evaluate the effects of steroidal or triterpenoidal sources of saponins from phytogenic extracts on in vitro CH_4_ production and ruminal fermentation. Additionally, saponin sources were evaluated at multiple inclusion levels that resemble practical feeding conditions or supranutritional inclusion. The experimental design was a randomized complete block design with 7 treatments in a 2 × 3 + 1 factorial arrangement of treatments. Two liquid sources of saponins containing 50% solids were tested: YSE (Micro-Aid Liquid; DPI Global, Porterville, CA, USA) and *Quillaja saponaria* extract (QSE; Phytogenic Patch Plus Triple P; DPI Global, Porterville, CA, USA). There were three levels for each saponin source: 1X, 10X, and 20X. The 1X, 10X, and 20X treatments were 100 mg/kg substrate, 1000 mg/kg substrate, and 2000 mg/kg substrate, respectively. Treatments were corrected for active ingredient percentages of the premix. The basal substrate without any feed additive (0X) was included as a negative control.

Treatments were added to the fermentation vessels according to the proportions described in Table 2. Saponin treatments were prepared as 0.25%, 2.5%, or 5% (*v*/*v*) solutions diluted in water for the 1X, 10X, and 20X treatments, respectively. All vessels received 2 mL of water or added treatment diluted in water so that all flasks contained the same liquid volume. There were two replicate fermentation vessels for each treatment, and the experiment was replicated on four separate days.

#### 2.3.3. Fermentation Preparation

Conditions of the in vitro gas production protocol were similar to those described previously [43,44,45,46,47]. The same four ruminally-cannulated Holstein steers (initial BW = 554 ± 30.9 kg) used in experiment 2 were housed outdoors in dry lot pens (2.4 m × 14.6 m). Steers were fed the same high-concentrate diet used in experiments 1 and 2, and the diet was fed ad libitum once per day. Ruminal contents (1000 g) were collected 4 h after feeding from each steer and combined into an insulated container (YETI Rambler One Gallon Water Jug; Yeti Holdings, Inc., Austin, TX, USA) for transport to the laboratory. Ruminal contents were blended under CO_2_ headspace for 30 s and then squeezed through four layers of cheesecloth. Squeezed ruminal fluid (600 mL) was combined with buffer, macromineral, micromineral, and reducing solutions (total = 2700 mL) that were prepared as described by Goering and Van Soest [44]. Fermentation vessels used in the current study were 250 mL coated glass bottles (Cat. #7056; ANKOM Technology, Macedon, NY, USA). Samples of the basal diet were dried at 55 °C for 24 h and ground to pass a 2 mm screen for use as the substrate for the in vitro ruminal fermentation. Substrates (501 ± 0.281 mg) were pre-weighed into fermentation vessels and combined with 2 mL of water or liquid treatment diluted in water to aid in the dispersal of the buffered inoculum. The buffered inoculum (103.2 ± 2.52 g) was then added to each fermentation vessel. Fermentation vessels were gassed with CO_2_ for 20 s, capped with RF1 gas production modules (ANKOM Technology, Macedon, NY, USA), and placed into a 39 °C circulating water bath (89 L; Precision 2868 Circulating Water Bath; ThermoFisher Scientific Inc., Beverly, MA, USA) to equilibrate. After all fermentation vessels were added to the water bath, valves of the gas production modules were opened simultaneously to release any accumulated pressure. Then, valves were closed, and cumulative gas pressure was measured in 5 min intervals over a 24 h incubation period using ANKOM Gas Pressure Monitor software (version 11.4; ANKOM Technology, Macedon, NY, USA).

#### 2.3.4. Sample Collection and Analysis

At the completion of the 24 h incubation period, vessels were removed from the water bath and transferred to an ice bath to stop the fermentation. Valves of the RF1 gas production modules were opened and samples of gas from the headspace of each vessel were collected with a syringe and stored in serum tubes (BD Vacutainer; Beckton, Dickinson and Company, Franklin Lakes, NJ, USA). Gas samples were analyzed for CH_4_ concentration by gas chromatography (Agilent 7890A GC; Agilent Technologies, Santa Clara, CA, USA). Each gas sample (50 µL) was manually injected into the inlet with a small hub removable needle (7784-06; Hamilton Company, Reno, NV, USA) in a 100 µL Teflon syringe (7656-01; Hamilton Company, Reno, NV, USA). The samples were carried to the fused silica capillary column (19091J-413; HP-5 GC Column; Agilent Technologies, Santa Clara, CA, USA) with He at a 20:1 split ratio. Inlet, oven, and detector temperatures were set to 200 °C, 150 °C, and 250 °C, respectively. Upon column exit, separated CH_4_ was detected with a flame ionization detector and quantified using electronic integration. Methane concentrations were determined in reference to analytical standards composed of 1% (*v*/*v*) and 10% (*v*/*v*) CH_4_ balanced with N_2_ (American Welding & Gas, Lexington, KY, USA). The RF1 gas production modules were opened, pH of the fermentation media was measured using a combination electrode and meter (SevenCompact S220; Mettler-Toledo International Inc., Columbus, OH, USA), and a 1 mL aliquot of the fermentation media was prepared and analyzed for VFA and NH_3_ concentrations as described in experiment 2.

#### 2.3.5. In Vitro Gas Production

The cumulative gas pressure was corrected for atmospheric pressure, converted to moles of gas produced using the ideal gas law, and then converted to milliliters of gas produced under standard conditions using Avogadro’s law. Cumulative gas production was corrected for the gas volume in the headspace of each fermentation vessel. The gas volume in the headspace of each fermentation vessel was calculated as the total volume of the vessel (308.2 ± 5.22 mL) minus the sum of the volume of the buffered inoculum and substrate (103.8 ± 2.47 mL). Cumulative gas production was fitted to the exponential model described by Pitt, et al. [48] using GraphPad Prism 5 (Dotmatics, Boston, MA, USA):

*F*(*t*) = 1 − e^−*r*(*t* − *λ*)^ (4)

where *F*(*t*) is the cumulative gas production, *r* is the rate of gas production, *t* is the time in hours, and *λ* is the lag time in hours. The rate of gas production was converted to a percentage by multiplying by 100. Methane production was determined by multiplying the CH_4_ concentration of gas samples by cumulative gas production after 24 h of fermentation.

### 2.4. Statistical Analysis

For experiment 1, variance–covariance structures for the statement of the repeated measure were assessed for fit using Bayesian information criterion for antedependence 1, autoregressive 1, compound symmetry, simple, and unstructured, including the steer as the subject. Whole-body O_2_ consumption, CO_2_ production, and CH_4_ production were analyzed using the repeated measures statement of the MIXED procedure of SAS for fixed effects of replicate, day, treatment, period, and their interactions. The initial measurements of O_2_ consumption, CO_2_ production, and CH_4_ production on day 8 were included in the model statements as covariates.

For experiment 2, ruminal Cr concentrations were fitted to the nonlinear equation previously described using the NLIN procedure of SAS. The in situ ruminal DM degradation data were analyzed using the NLIN procedure of SAS to estimate the parameters of the equation previously described. Degradation parameters and liquid passage characteristics were analyzed using the GLM procedure of SAS for fixed effects of period, treatment, and the period × treatment interaction. Variance–covariance structures were tested for ruminal VFA, NH_3_, and L(+)-lactate concentrations as described in experiment 1. Concentrations of ruminal fermentation end-products were analyzed using the repeated measures statement of the MIXED procedure of SAS for fixed effects of period, time, treatment, and the time × treatment interaction. The initial metabolite concentration (0 h) was included in the model statement as a covariate for ruminal metabolites.

For experiment 3, gas production kinetics and fermentation end-products were analyzed using the GLM procedure of SAS for fixed effects of the replicate and treatment. The IML procedure was used to generate orthogonal contrast coefficients to adjust for the unequal spacing between the treatment levels. Contrast statements were used to determine the differences between treatments. To determine the effect of anti-coccidial supplementation, a contrast was analyzed for basal substrate vs. others. To determine the effect of ionophore supplementation (antibiotic vs. non-antibiotic anti-coccidials), a contrast was analyzed for monensin vs. DCQ and amprolium. To determine the effect of the non-antibiotic anti-coccidial source, a contrast was analyzed for DCQ vs. amprolium. For each anti-coccidial source (monensin, DCQ, amprolium), a linear polynomial contrast (0X, 1X, 10X) was analyzed to determine the effects of supplementation level.

For experiment 4, gas production kinetics and fermentation end-products were analyzed using the GLM procedure of SAS for the fixed effects of the replicate and treatment. The IML procedure was used to generate orthogonal contrast coefficients to adjust for unequal spacing between treatment levels. Contrast statements were used to determine differences between treatments. To determine the effect of saponin supplementation, a contrast was analyzed for basal substrate vs. the others. To determine the effect of the saponin source, a contrast was analyzed for YSE vs. QSE. For each saponin source, linear and quadratic polynomial contrasts (0X, 1X, 10X, 20X) were analyzed to determine the effects of supplementation level.

All data were checked for normality using the Shapiro–Wilk test of the UNIVARIATE procedure of SAS. Blocks (replicates) were included in the model statement as fixed effects, as recommended by Dixon [49]. Pairwise differences of least squares means were separated using the Tukey–Kramer adjustment, protected by a significant F-test. Results were considered significant if *p* ≤ 0.05. Tendencies were declared when 0.05 < *p* ≤ 0.10.

## 3. Results

### 3.1. Experiment 1

Supplementation of DCQ + YSE did not influence whole-body O_2_ consumption and the CO_2_ or CH_4_ production of steers fed a high-concentrate diet (Table 3). The respiratory quotient was not different between dietary treatment groups.

### 3.2. Experiment 2

Supplementation of DCQ + YSE did not influence the soluble or potentially degradable DM fractions of the basal diet (Table 4). The potential rate and extent of DM degradation were not influenced by DCQ + YSE supplementation. The in situ ruminal DM degradability of the basal diet was not affected by dietary treatments. The fractional rate of liquid passage, ruminal liquid retention time, rumen liquid volume, and ruminal liquid outflow were not influenced by DCQ + YSE supplementation.

The combination of DCQ + YSE supplementation increased (*p* = 0.03) ruminal L(+)-lactate concentration (Table 5). Ruminal NH_3_ and total VFA concentrations were not influenced by DCQ + YSE supplementation. The ruminal acetate molar proportion and acetate:propionate were not changed by DCQ + YSE supplementation. Supplementation of DCQ + YSE tended to increase (*p* = 0.09) ruminal propionate proportion. Molar proportions of isobutyrate and butyrate were not influenced by DCQ + YSE supplementation. Supplementation of DCQ + YSE decreased (*p* = 0.01) ruminal isovalerate proportion and increased (*p* < 0.01) ruminal valerate proportion.

### 3.3. Experiment 3

Increasing monensin inclusion linearly increased (*p* < 0.01) pH of the fermentation media after 24 h of incubation (Table 6). The inclusion of anti-coccidial compounds increased (*p* = 0.02) gas production after 24 h, but gas production was greater (*p* < 0.01) for DCQ and amprolium compared with monensin. Gas production decreased (*p* < 0.01) linearly with increasing monensin inclusion, increased (*p* < 0.01) linearly with DCQ inclusion, and tended to increase (*p* = 0.07) linearly with amprolium inclusion. Monensin inclusion produced a faster (*p* < 0.01) rate of gas production compared with DCQ and amprolium. Increasing the monensin inclusion linearly increased the rate of gas production. Increasing DCQ and amprolium inclusion linearly decreased (*p* < 0.01) and tended to decrease (*p* = 0.07) the rate of gas production. Monensin inclusion decreased (*p* < 0.01) CH_4_ percentage and production compared with DCQ and amprolium. Increasing monensin linearly decreased (*p* < 0.01) CH_4_ percentage and production. Ammonia concentration linearly decreased (*p* = 0.04) with increasing DCQ inclusion.

Total VFA concentrations were lower (*p* = 0.05) for monensin compared with DCQ and amprolium. Acetate molar proportion linearly decreased (*p* = 0.02) with increasing levels of DCQ. Propionate proportion was greater (*p* < 0.01) for all treatments containing anti-coccidial compounds compared to the basal substrate. However, the propionate proportion was greater (*p* < 0.01) when monensin was included in the in vitro fermentation compared with DCQ and amprolium. Increasing monensin and DCQ inclusion linearly increased (*p* < 0.01) the molar proportion of propionate. Isobutyrate and butyrate molar proportions were greater (*p* ≤ 0.04) for non-antibiotic anti-coccidial compounds compared with monensin. Increasing monensin inclusion linearly decreased (*p* < 0.01) butyrate proportion and tended to decrease (*p* = 0.08) isobutyrate proportion linearly. Isobutyrate proportion tended to increase (*p* = 0.08) linearly with increasing inclusion of DCQ. The isovalerate proportion was greater (*p* < 0.01) with monensin inclusion compared with DCQ and amprolium because increasing monensin inclusion linearly increased (*p* < 0.01) isovalerate proportion. Valerate proportion linearly decreased (*p* = 0.04) with monensin inclusion and linearly increased (*p* = 0.04) with amprolium inclusion. The anti-coccidial compound inclusion decreased (*p* < 0.01) the acetate:propionate, with the acetate:propionate being reduced (*p* < 0.01) to the greatest extent by monensin. Increasing levels of DCQ and monensin linearly decreased (*p* < 0.01) the acetate:propionate.

### 3.4. Experiment 4

The pH of the fermentation media after 24-h of incubation was greater (*p* < 0.01) with QSE inclusion compared with YSE (Table 7). Gas production after 24 h was greater (*p* < 0.01) for YSE compared with QSE. Increasing levels of YSE inclusion linearly increased (*p* = 0.02) gas production after 24 h. The rate of gas production was greater (*p* < 0.01) for QSE compared with YSE. Increasing levels of QSE inclusion linearly increased (*p* < 0.01) the rate of gas production. Methane percentage and production were not influenced by saponin inclusion. Ammonia and total VFA concentrations were not influenced by saponin inclusion.

The inclusion of saponins decreased (*p* = 0.04) acetate proportion. Increasing YSE and QSE inclusion decreased (*p* < 0.01) and tended to decrease (*p* = 0.06) acetate proportion linearly, respectively. Increasing YSE and QSE inclusion linearly increased (*p* < 0.01) propionate proportion. Increasing YSE and QSE inclusion linearly decreased (*p* ≤ 0.04) the molar proportions of isobutyrate, butyrate, and isovalerate. Butyrate proportion was greater (*p* = 0.03) in fermentation media with YSE inclusion compared with QSE. Increasing YSE and QSE inclusion linearly increased (*p* < 0.01) valerate proportion; however, the valerate proportion was greater (*p* < 0.01) with QSE inclusion. Increasing YSE and QSE inclusion linearly decreased (*p* < 0.01) the acetate:propionate.

## 4. Discussion

### 4.1. Effects of DCQ + YSE on In Vivo Ruminal Fermentation and CH_4_ Production

Previous research demonstrated that YSE supplementation decreased in vitro CH_4_ production across substrates that contained low-, medium-, or high-proportions of roughages [14]. In the current study, supplementation of DCQ + YSE for up to 10 d did not influence in vivo CH_4_ production of steers. Because YSE supplementation had previously resulted in reduced CH_4_ production in vitro [14], we assumed that 7 d of adaptation would be adequate time to allow for ruminal turnover to observe effects on CH_4_ production. The liquid retention time measured in experiment 2 (18.5–19.9 h) suggests that there was multiple ruminal turnovers within the 7 d adaptation period. However, it is possible that longer adaptation to dietary treatments was necessary to observe effects on CH_4_ production. Also, feeding different sources and levels of DCQ and YSE and variation in the concentration of saponins and/or types of steroidal saponins present in YSE could potentially alter responses in CH_4_ production.

Reductions in CH_4_ production could potentially be associated with increases or decreases in economically important variables in cattle [2]. For example, decreased CH_4_ production could be due to decreased intake, decreased digestibility, or decreased VFA production and, therefore, could negatively affect production outcomes [2]. In contrast, decreased CH_4_ production associated with decreased protozoa, greater propionate production, and/or increased energy retention could be beneficial for both productive and environmental outcomes [2]. Feed intake was controlled by limiting energy intake to two times NE_m_ in experiments 1 and 2. Results from the in situ degradability experiment demonstrated that the rate and extent of DM degradation and the in situ ruminal DM degradability of the basal diet were not affected by DCQ + YSE supplementation. Likewise, ruminal liquid passage kinetics were not affected by DCQ + YSE supplementation.

Changes in end-products of ruminal fermentation in experiment 2 may suggest that supplementation of DCQ + YSE modified ruminal fermentation similarly when compared to previous studies. In general, supplementation of YSE typically results in decreased ruminal NH_3_ concentration, increased ruminal propionate proportion, and decreased ruminal protozoa concentration [50]. Although not statistically significant, ruminal NH_3_ concentration was numerically reduced by 9.15% with DCQ + YSE supplementation. Ruminal propionate proportion tended to increase with DCQ + YSE supplementation. Although there was a tendency for a numerical increase in the propionate proportion, DCQ + YSE supplementation did not influence the acetate proportion or the acetate:propionate. It should be noted that the acetate:propionate was 1.43 and 1.41 for control and DCQ + YSE treatments, respectively. The decreased acetate proportion and increased propionate proportion result in a decreased acetate:propionate is closely correlated with decreased CH_4_ production [51]. It is possible that the current diet composition and feeding level were propiogenic, limiting the opportunity for YSE to shift H_2_ sinks. A recent review pointed out that the positive effects of YSE supplementation are not always observed when included in diets for cattle [50]. Sources of saponin-containing extracts, plant saponin composition, dietary inclusion levels, manufacturing processes, and interactions with dietary components are some factors that could potentially contribute to inconsistencies across studies [14].

### 4.2. Effects of Monensin on In Vitro Ruminal Fermentation and CH_4_ Production

A recent meta-analysis reported that monensin supplementation decreased CH_4_ production in beef steers and dairy cows [52]. Of the anti-coccidial and saponin sources tested, only monensin decreased in vitro CH_4_ production in the current study. Monensin decreased total VFA concentrations and gas production in the current study, which is similar to the findings of others using in vitro gas production systems [53]. However, decreased total VFA concentration is not typically observed when cattle are fed monensin [54]. Rather, monensin can alter ruminal microbial populations by decreasing protozoa and Gram-positive bacteria, resulting in less acetate, butyrate, CH_4_, lactate, and NH_3_ production [55,56]. Consistent with numerous previous in vitro and in vivo studies, monensin inclusion decreased the acetate:propionate and increased molar propionate proportion [53,57,58,59,60]. Decreased isobutyrate and valerate proportions with monensin inclusion are likely due to the anti-microbial effects of monensin on proteolytic and/or amino acid-fermenting bacteria [61], suggesting reduced amino acid degradation [62]. Decreased butyrate proportion with monensin inclusion was also found previously when a concentrate substrate was used [53]. In the current study, monensin did not influence NH_3_ concentration which contrasts with several studies reporting that monensin decreased NH_3_ concentration [53,61,63]. The lack of effect of monensin on NH_3_ concentration in the current study could be due to the excess N available from the in vitro buffer solutions [44], as well as, the basal substrate which exceeded requirements for ruminally degradable protein. Overall, monensin modified in vitro ruminal fermentation and CH_4_ production consistent with several previous experiments [53,64,65,66,67].

### 4.3. Effects of Non-Antibiotic Anti-Coccidial Compounds on In Vitro Ruminal Fermentation and CH_4_ Production

In contrast to monensin, non-antibiotic anti-coccidial compounds had minimal impacts on in vitro CH_4_ production and ruminal fermentation. Feeding increasing levels of DCQ did not influence the total-tract DM digestibility of steers fed a high-concentrate diet [22]. In contrast, results from the current experiment demonstrated that increasing levels of DCQ linearly increased in vitro gas production. However, this is likely due to changes in the fermentability of the Deccox premix, which replaced the basal substrate (2.1% and 20.1% inclusion rate for 1X and 10X treatments) in the current experiment. According to the manufacturer, the Deccox premix also contained corn meal, soybean oil, lecithin, and silicone dioxide. It is possible that some of the increase in propionate proportion in experiment 2 with DCQ + YSE supplementation could have been due to both DCQ and YSE, as increasing DCQ linearly increased in vitro propionate proportion in experiment 3. Whether or not those effects are due to DCQ or the premix itself remains to be determined. Similar to previous in vivo findings, DCQ had little or no influence on characteristics of ruminal fermentation, including VFA profiles or CH_4_ production in the current study. Like DCQ, amprolium inclusion had minimal effects on in vitro ruminal fermentation in the current study.

### 4.4. Effects of YSE and QSE on In Vitro Ruminal Fermentation and CH_4_ Production

Previous research had demonstrated that YSE inclusion decreased in vitro CH_4_ production across 10%, 50%, and 100% forage-based diets [14]. However, increasing YSE or QSE did not influence in vitro CH_4_ production in the current experiment. In the current study, increasing levels of saponins linearly decreased acetate proportion and increased propionate proportion, resulting in decreased acetate:propionate. Changes in molar propionate proportion with an absence of a change in in vitro CH_4_ production in experiment 4 are similar to the results found in experiments 1 and 2, where the combination of DCQ + YSE tended to increase the molar proportion of propionate without influencing CH_4_ production. Zúñiga-Serrano, et al. [50] proposed that YSE may modify ruminal fermentation through several mechanisms which can lead to downstream effects on enteric CH_4_ production. *Yucca schidigera* extract or saponins from YSE can decrease ruminal cellulolytic bacteria and fungi [68], decrease methanogenic archaea [69], and decrease ruminal protozoa [13,15,70]. Reduced ruminal NH_3_, decreased acetate:propionate, and decreased fiber degradation are associated with decreased CH_4_ production with YSE inclusion [50]. One study found that YSE and QSE decrease CH_4_ production but at much greater concentrations than those used in the current experiment [13]. Sources of saponin-containing extracts, plant saponin composition, dietary inclusion levels, manufacturing processes, and interactions with dietary components are some factors that could potentially contribute to inconsistencies across studies [14].

## 5. Conclusions

Supplementation of DCQ + YSE for 7 to 10 days did not influence O_2_ consumption, CO_2_ production, or CH_4_ production in steers consuming a high-concentrate diet at 2 × NE_m_. Supplementation of DCQ + YSE did not influence the rate or extent of ruminal DM degradation of the basal finishing diet or liquid passage kinetics. Supplementation of DCQ + YSE did not influence total VFA concentrations but tended to increase ruminal propionate proportion. Increasing levels of monensin decreased in vitro CH_4_ production, and acetate:propionate, isovalerate, and valerate proportions. Decoquinate and amprolium had minimal effects on in vitro ruminal fermentation. Increasing YSE or QSE inclusion increased propionate proportion but was not accompanied by a reduction in in vitro CH_4_ production. Further research is necessary to identify alternative non-antibiotic compounds for the simultaneous reduction of CH_4_ emissions and control of coccidiosis in feedlot cattle diets.

## Figures and Tables

**Table 1:** Composition of the high-concentrate diet fed to steers in experiments 1–4.

Item	
Ingredient composition, DM basis	
Cracked corn, %	27.5
High-moisture corn, %	27.5
Dried corn distillers’ grains with solubles, %	25.0
Corn silage, %	10.0
Fine-ground corn, %	6.95
Limestone, %	1.92
Trace mineral premix, % ^1^	0.50
Urea, %	0.36
Choice white grease, %	0.25
Vitamin A, D, and E premix, % ^2^	0.02
Chemical composition	
Dry matter, %	71.5
Crude protein, % of DM	14.5
Neutral detergent fiber, % of DM	12.1
Acid detergent fiber, % of DM	6.7
Ca, % of DM	0.73
P, % of DM	0.45
Net energy for maintenance, Mcal/kg ^3^	1.95
Net energy for gain, Mcal/kg ^3^	1.30

^1^ Contained: 56.34% Cl, 36.53% Na, 1.2% S, 0.06% Ca, 9.29 g Fe/kg, 5.52 g Zn/kg, 4.79 g Mn/kg, 1.84 g Cu/kg, 120 mg I/kg, 68.9 mg Co/kg, and 18.5 mg Se/kg on a DM basis. ^2^ Composed of vitamin A acetate (1814 kIU/kg), D-activated animal sterol (source of vitamin D_3_; 363 kIU/kg), vitamin E supplement (227 IU/kg), roughage products, calcium carbonate, and mineral oil. ^3^ Calculated from tabular values [25].

**Table 2:** Description of treatments used in experiments 3 and 4.

	Source	Level	Amt. Basal Substrate	Amt. Mix Substrate	Liquid Added ^4^
Exp. 3					
	Basal substrate (control)	0X	500 mg	-	Water
	Monensin	1X	496.25 mg	3.75 mg of premix ^1^	Water
	Monensin	10X	462.5 mg	37.5 mg of premix ^1^	Water
	Decoquinate	1X	489.6 mg	10.4 mg of premix ^2^	Water
	Decoquinate	10X	396 mg	104 mg of premix ^3^	Water
	Amprolium	1X	500 mg	-	Corid 9.6% Oral solution
	Amprolium	10X	500 mg	-	Corid 9.6% Oral solution
Exp. 4					
	Basal substrate (control)	0X	500 mg	-	Water
	*Yucca schidigera* extract	1X	500 mg	-	0.25% Micro-Aid Liquid solution
	*Yucca schidigera* extract	10X	500 mg	-	2.5% Micro-Aid Liquid solution
	*Yucca schidigera* extract	20X	500 mg	-	5% Micro-Aid Liquid solution
	*Quillaja saponaria* extract	1X	500 mg	-	0.25% Phytogenic Patch Plus solution
	*Quillaja saponaria* extract	10X	500 mg	-	2.5% Phytogenic Patch Plus solution
	*Quillaja saponaria* extract	20X	500 mg	-	5% Phytogenic Patch Plus solution

^1^ Rumensin 90 premix was mixed with the basal substrate on a 10% (*w*/*w*) basis. ^2^ Deccox premix was mixed with the basal substrate on a 10% (*w*/*w*) basis for the 1X treatment. ^3^ Deccox premix was mixed with the basal substrate on a 50% (*w*/*w*) basis for the 10X treatment. ^4^ Two milliliters of each solution were added to their respective fermentation vessels. Corid 9.6% Oral Solution was diluted with 6.4 mL/100 mL and 64 mL/100 mL for the 1X and 10X treatments, respectively. Liquid saponin treatments were mixed with water on a *v/v* basis.

**Table 3:** Experiment 1: The effects of DCQ + YSE supplementation on in vivo daily O_2_ consumption and CO_2_ and CH_4_ production of steers limit-fed a high-concentrate diet.

	Treatment			
Item	Control	DCQ + YSE	SEM ^1^	*p*-Value
O_2_ consumption				
L	2963	2931	66.9	0.74
L/kg DMI	419	413	10.1	0.71
L/kg BW	8.95	8.90	0.193	0.85
L/kg BW^0.75^	38.2	37.9	0.81	0.82
CO_2_ production				
L	3009	2964	68.7	0.65
L/kg DMI	425	418	10.5	0.63
L/kg BW	9.09	9.00	0.197	0.75
L/kg BW^0.75^	38.7	38.3	0.83	0.72
CH_4_ production				
L	98.6	94.3	6.43	0.64
L/kg DMI	13.8	13.2	0.91	0.62
L/kg BW	0.294	0.281	0.0172	0.60
L/kg BW^0.75^	1.26	1.20	0.075	0.61
Respiratory quotient	1.01	1.01	0.004	0.64

Abbreviations: BW, body weight; BW^0.75^, metabolic body weight; DMI, dry matter intake. ^1^ Standard error of the mean (*n* = 8).

**Table 4:** Experiment 2: The effects of DCQ + YSE supplementation on in situ ruminal DM degradation kinetics and liquid passage rate of steers limit-fed a high-concentrate diet.

	Treatment			
Item	Control	DCQ + YSE	SEM ^1^	*p*-Value
Soluble fraction, %	53.1	51.6	0.78	0.24
Potentially degradable fraction, %	29.9	31.9	1.34	0.35
Potential extent of degradation, %	83.0	83.4	0.98	0.74
Lag time, h	4.24	2.72	2.81	0.72
Fractional rate of degradation, % per h	3.08	3.22	0.489	0.85
Fractional rate of liquid passage, % per h	5.52	5.23	0.546	0.72
In situ ruminal degradability, %	63.6	63.7	0.96	0.99
Liquid retention time, h	18.5	19.9	1.83	0.61
Rumen liquid volume, L	30.0	29.2	6.46	0.94
Rumen liquid outflow, L/h	1.60	1.46	0.305	0.77

^1^ Standard error of the mean (*n* = 4).

**Table 5:** Experiment 2: The effects of DCQ + YSE supplementation on ruminal fermentation characteristics of steers limit-fed a high-concentrate diet.

	Treatment			
Item	Control	DCQ + YSE	SEM ^1^	*p*-Value
L(+)-Lactate, mM	0.545	1.32	0.250	0.03
NH_3_, mM	6.67	6.06	0.289	0.14
Total VFA, mM	125	119	3.8	0.33
mol/100 mol				
Acetate	46.8	45.8	0.49	0.19
Propionate	33.7	34.9	0.51	0.09
Isobutyrate	0.524	0.468	0.0607	0.12
Butyrate	10.8	10.1	0.36	0.18
Isovalerate	2.12	1.80	0.082	0.01
Valerate	5.57	7.43	0.239	<0.01
Acetate:propionate	1.43	1.41	0.031	0.64

^1^ Standard error of the mean (*n* = 4).

**Table 6:** Experiment 3: The effects of sources and levels of anti-coccidial compounds on in vitro CH_4_ production and ruminal fermentation.

		Treatment							Contrast *p*-Value ^2^					
Item	0X	MON		DCQ		AMP				Source		Level		
		1X	10X	1X	10X	1X	10X	SEM ^1^	ACC	IPH	DCQ vs. AMP	Linear MON	Linear DCQ	Linear AMP
pH	6.78	6.81	6.87	6.84	6.83	6.85	6.83	0.014	<0.01	0.92	0.8	<0.01	0.2	0.34
Gas production, mL	116	111	93.5	115	122	115	120	1.4	0.02	<0.01	0.42	<0.01	<0.01	0.07
Rate, %/h	13.7	15	16	14	11.6	13.8	12.7	0.42	0.8	<0.01	0.3	<0.01	<0.01	0.07
CH_4_, %	7.83	7.43	5.79	7.75	7.92	7.76	7.68	0.252	0.11	<0.01	0.65	<0.01	0.74	0.66
CH_4_, mL	9.14	8.46	5.51	9.09	9.84	9.02	9.27	0.299	0.07	<0.01	0.3	<0.01	0.11	0.6
NH_3_, mM	27.5	27.3	25.7	27.2	25.3	27.2	26.3	0.83	0.25	0.97	0.58	0.19	0.04	0.19
Total VFA, mM	61.5	57	60.7	62.5	64.5	60.7	64.8	2.48	0.92	0.05	0.73	0.64	0.24	0.34
mol/100 mol														
Acetate	41.6	41.3	41.5	41.3	40.8	41.1	41	0.31	0.24	0.21	0.99	0.93	0.02	0.17
Propionate	18	18.8	21.1	18.5	18.6	18.6	18.4	0.24	<0.01	<0.01	0.8	<0.01	<0.01	0.32
Isobutyrate	5.28	5.15	4.58	5.25	5.38	5.21	5.11	0.208	0.47	0.04	0.47	0.08	0.08	0.16
Butyrate	12.6	12.1	10.4	12.4	12.6	12.4	12.6	0.23	0.04	<0.01	0.99	<0.01	0.64	0.4
Isovalerate	5.43	5.52	5.68	5.4	5.35	5.45	5.45	0.045	0.39	<0.01	0.1	0.01	0.12	0.85
Valerate	5.61	5.73	5.4	5.59	5.64	5.64	5.76	0.065	0.82	0.14	0.15	0.04	0.78	0.04
Acetate:propionate	1.92	1.82	1.58	1.85	1.83	1.84	1.86	0.019	<0.01	<0.01	0.79	<0.01	<0.01	0.19

Abbreviations: ACC, anti-coccidial compound; AMP, amprolium; DCQ, decoquinate; IPH, ionophore; MON, monensin. ^1^ Standard error of the mean (*n* = 8). ^2^ Contrasts: ACC = 0X vs. others; IPH (antibiotic vs. non-antibiotic anti-coccidials) = MON vs. DCQ and AMP; DCQ vs. AMP = decoquinate vs. amprolium.

**Table 7:** Experiment 4: The effects of sources and levels of phytogenic saponin extracts on in vitro CH_4_ production and ruminal fermentation.

		Treatment							Contrast *p*-Value ^2^					
Item	0X	YSE			QSE				SAP	YSE vs. QSE	Level			
		1X	10X	20X	1X	10X	20X	SEM ^1^			Lin. YSE	Quad. YSE	Lin. QSE	Quad. QSE
pH	6.88	6.88	6.88	6.87	6.90	6.90	6.89	0.008	0.20	<0.01	0.67	0.34	0.75	0.16
Gas production, mL	114	116	116	120	111	108	114	2.2	0.96	<0.01	0.02	0.43	0.88	0.08
Rate, %/h	12.9	12.7	12.8	12.9	12.5	13.9	15.4	0.45	0.39	<0.01	0.89	0.82	<0.01	0.80
CH_4_, %	7.67	7.71	7.24	7.25	7.49	7.47	7.58	0.234	0.39	0.56	0.13	0.44	0.91	0.61
CH_4_, mL	8.78	8.97	8.34	8.72	8.30	8.03	8.53	0.313	0.38	0.13	0.59	0.28	0.75	0.13
NH_3_, mM	27.2	25.9	27.1	26.8	27.4	28.4	26.8	0.93	0.91	0.22	0.76	0.83	0.86	0.22
Total VFA, mM	66.8	64.3	67.6	69.7	63.3	67.3	67.3	2.20	0.93	0.52	0.11	0.81	0.42	0.81
mol/100 mol														
Acetate	47.4	47.3	47.1	46.5	46.5	47.0	46.7	0.24	0.04	0.26	<0.01	0.31	0.06	0.51
Propionate	24.0	24.2	24.9	25.2	24.6	24.9	25.6	0.24	<0.01	0.17	<0.01	0.30	<0.01	0.96
Isobutyrate	2.03	2.03	1.99	1.95	2.04	2.02	1.94	0.016	0.03	0.46	<0.01	0.87	<0.01	0.11
Butyrate	14.9	14.8	14.4	14.6	14.7	14.4	14.0	0.13	<0.01	0.03	0.04	0.07	<0.01	0.73
Isovalerate	4.72	4.70	4.63	4.55	4.71	4.69	4.55	0.043	0.07	0.47	<0.01	0.85	<0.01	0.27
Valerate	6.94	6.95	6.96	7.23	6.98	7.29	7.32	0.069	0.01	<0.01	<0.01	0.09	<0.01	0.07
Acetate:propionate	2.01	2.00	1.92	1.87	1.95	1.91	1.84	0.027	<0.01	0.15	<0.01	0.51	<0.01	0.75

Abbreviations: SAP, saponins; QSE, *Quillaja saponaria* extract; YSE, *Yucca schidigera* extract. ^1^ Standard error of the mean (*n* = 8). ^2^ Contrasts: Saponin = 0X vs. others; YSE vs. QSE = *Yucca schidigera* extract vs. *Quillaja saponaria* extract.

## Data Availability

Data will be made available without reservation upon request to the corresponding author.
